# Prevalence of multiresistant gram-negative organisms in a tertiary hospital in Mwanza, Tanzania

**DOI:** 10.1186/1756-0500-2-49

**Published:** 2009-03-26

**Authors:** Stephen E Mshana, Erasmus Kamugisha, Mariam Mirambo, Trinad Chakraborty, Eligius F Lyamuya

**Affiliations:** 1 Weill Bugando University College of Health Science Box 1464, Mwanza, Tanzania; 2 Institute of Medical Microbiology Giessen, Frankfurter strasse 107, 35392 Giessen, Germany; 3 Departments of Microbiology and Immunology, Muhimbili University of Health and Allied Sciences, PO Box 65001 Dar-es-Salaam, Tanzania

## Abstract

**Background:**

Antimicrobial resistance is fast becoming a global concern with rapid increases in multidrug-resistant Gram negative organisms. The prevalence of extended spectrum beta-lactamase (ESBL)-producing clinical isolates increases the burden on implementing infectious disease management in low socio-economic regions. As incidence can vary widely between regions, this study was done to determine resistance patterns of Gram-negative organisms at Bugando Medical Center, a tertiary hospital in Mwanza, Tanzania.

**Methods:**

A total of 800 clinical samples (urine, wound swab, pus, blood, aspirate, sputum etc) were processed over a period of 6 months. Gram-negative bacteria were identified using conventional in-house biochemical tests and susceptibility to common antibiotics done using disc diffusion methods. The disc approximation method was used to identify ESBL producers.

**Results:**

A total of 377 Gram-negative bacteria (GNB) recovered from 377 clinical specimens were analyzed of which 76.9% were Enterobacteriaceae. Among all GNB, 110/377 (29.2%) were found to be ESBL producers. Species specific ESBLs rate among *Klebsiella pneumoniae, Escherichia coli*, *Acinetobacter spp*, *Proteus spp *and other enterobacteria were 63.7%, 24.4%, 17.7%, 6.4% and 27.9% respectively. A statistically significant higher number of inpatients 100/283 (35.3%) compared to 10/94 (10.6%) of outpatients had ESBL-producing organisms (p = 0.000023). Rates of resistances to gentamicin, tetracycline, sulphamethaxazole/trimethoprim and ciprofloxacin were significantly higher among ESBLs isolates than non-ESBL isolates (p = 0.000001).

**Conclusion:**

ESBL producing organisms are common at BMC (Bugando Medical Center) and pose a challenge to antibiotic therapy. Successful implementation of a routine detection of ESBL production is essential in designing appropriate antibiotic prescribing policies and infection control intervention programmes.

## Background

Antimicrobial resistance among enteric Gram negative bacteria is fast becoming a global public health concern with rapid increase in multidrug resistant organisms [1]. Gram negative bacteria (GNB) are a common cause of urinary tract infections, neonatal sepsis and post surgical infections in hospitalized patients [1,2]. Resistance of Enterobacteriaceae to broad spectrum β-lactam antibiotics via ESBL production is an increasing problem worldwide [2].

The prevalence of ESBL producing clinical isolates is more than 20% in Asia and South Africa [3]. ESBLs have been found in 30 to 60% of klebsiellae from intensive care unit in Brazil, Columbia and Venezuela [4-6]. In North America, National Nosocomial Infection Surveillance revealed that 6.1% of *Klebsiella pneumoniae *from 110 intensive care units were resistant to third generation cephalosporins [7]. There is considerable geographical variation in the occurrence of ESBLs in European countries, with marked hospital to hospital differences within the countries [8]. In Tanzania there is little data on ESBLs epidemiology, in a study conducted recently at Muhimbili National Hospital more than 80% of isolates were reported to be resistant to ampicillin and 25% of *Escherichia coli *isolates were resistant to third generation cephalosporins [9,10]. Prevalence of ESBL-producing strains in various species of Enterobacteriaceae differs in different countries and in different hospitals. Usually one of three species (*Klebsiella pneumoniae, Escherichia coli*, *Enterobacter spp*) predominates [11].

Antimicrobial agents are the most important tool available for managing infectious diseases of bacterial origin. Some of ESBL are untreatable; an observation that reflects the formidable challenge that resistance producing strains can pose in terms of disease control and prevention [2]. The prevention of nosocomial infections and their transmission requires reliable microbiological diagnosis, rational antibiotic prescribing and effective infection control. The most important determinants in treating patients with infections in the ICU is prompt initiation of effective empirical antimicrobial therapy, taking note of the observation that inappropriate empirical therapy affects patient mortality rates [11].

It is therefore essential to address this issue as the cornerstone to prevent the emergence of multiresistant organisms. This study aimed at addressing the situation at Bugando Medical Centre in order to provide evidence of the existence and incidence of ESBL-producing bacteria. Bugando Medical Centre is a tertiary healthcare institution with 800 beds and serves a catchment area comprising 13 million people. This study underscores the need for having in place a routine surveillance of ESBLs and antimicrobial susceptibility pattern of isolates which are routinely encountered in clinical practice in this tertiary health care institution. In addition, it emphasizes the need for immediate strengthening of hospital-based infection prevention programmes through effective antimicrobial resistance surveillance.

## Methods

A total of 800 clinical samples obtained from 800 patients (male to female ratio of 1.4:1) were processed over a period of 6 months. These samples included; urine, wound swabs, pus, blood, aspirate and sputum. Sample size was estimated using Kish and Lisle formula of crossectional studies [9]. All specimens were delivered and processed as per standard operating procedures of the laboratory; all incubations were done aerobically for 18–24 hrs [12]. Gram negative bacteria were identified using colonial morphology on Blood and MacConkey agar (Oxoid UK) followed by simple biochemical panel (TSI, Citrate test, SIM, Urease, VP and Methyl red test); negative and positive controls were included in each test [12]. A total of 377 clinical specimens with single significant growth of Gram negative bacteria were included in the analysis.

Susceptibility testing was done using disc diffusion method; procedures were performed following guidelines laid down by the Clinical Laboratory Standard Institute (CLSI) [13]. *Escherichia coli *ATCC 25922 was used as quality control strain. The antibiotic discs representing common drugs used for treatment of suspected Gram negative bacterial infection in our hospital were tested, plus other reserve drugs for Gram negative bacteria commercially available in our setting. Discs included ampicillin (10 μg), amoxyillin/clavunate (20/10 μg), ciprofloxacin (5 μg), tetracycline (30 μg), gentamicin (10 μg), sulfamethoxazole/trimethoprim1.25/23.75 μg) and ceftriaxone. Other reserve discs included piperacillin/combactam (100/10 μg), ceftazidime (30 μg), cefepime (30 μg), imipenem (10 μg) and meropenem (10 μg) (Oxoid UK).

Test organisms were suspended in normal saline to 0.5 McFarland standard and then inoculated on Muller Hinton agar plates (Oxoid, Wade Road, Basingstoke UK), followed by overnight incubation at 37°C for 18–24 hrs. Interpretation was done using guidelines laid down in the CLSI manual, which provides break points corresponding to zone of inhibition diameter [13].

The isolates were screened for ESBL production using MacConkey agar with 30 μg/ml cefotaxime and confirmed using disc approximation method. Ceftazidime (30 μg) and cefotaxime (30 μg) discs were placed equidistant from the amoxycillin/clavunate (20/10 μg) disc; enhanced zone of inhibition towards amoxycillin/clavunate (20/10 μg) disc was considered as positive result for ESBL production [13-15]. Data were archived using Excel and SPSS programmes and all ESBL isolates were preserved for future molecular analysis.

The study was approved by BMC/WBUCH Ethics review board of Weill Bugando University College of Health Sciences. An informed consent was obtained before collection of appropriate specimens and results were used in the management of patients.

## Results

Three hundred and seventy seven non-duplicate Gram negative bacteria were isolated over a period of six months. The isolates included those from urine 156 (41.4%), wound swabs 147 (39.0%), blood 43 (11.4%), pus 22(5.8%), sputum 3(0.8%), bone chips 5 (1.3%) and aspirates 1(0.3%). Of 377 isolates, 290 (76.9%) were enterobacteriaceae.

Isolates recovered included, *Escherichia coli *(33.7%), *Klebsiella pneumoniae *(24.1%), *Pseudomonas spp *(12.2%), *Acinetobacter spp *(10.3%) and *Proteus spp *(8.2%). Other enterobacteria isolated were *Enterobacter spp *(2.9%), *Morganella morganii *(2.9%), *Klebsiella oxytoca *(2.3%), *Citrobacter spp *(1%), *Serratia spp *(0.8%), *Salmonella spp *(0.8%) and *Sternotrophomonas spp *(0.5%).

One hundred and ten isolates were confirmed to produce ESBL using disc approximation method indicating a prevalence of 29.2% ESBL production amongst all GNB as shown in table 1. Species specific ESBLs rate among *Klebsiella pneumoniae, Escherichia coli*, *Acinetobacter spp*, *Proteus spp *and other enterobacteria were 63.7%, 24.4%, 17.7%, 6.4% and 27.9% respectively p = 0.0001 (table 1). Among 283 isolates from inpatients, 100 (35.3%) were found to produce ESBL as compared to 10.6% of isolates from 94 outpatients as shown in table 1 (chi square = 22, p = 0.0001). Most of the isolates recovered from blood samples 37(86%) were ESBL producers compared to those from urine and wound swabs (chi square 79.76, p = 0.000001).

**Table 1:** ESBL producing organisms in different specimens and among inpatients and outpatients

**Isolate (n)**	**ESBL N (%)**	**Inpatients ESBL %**	**Outpatients ESBL %**
***Escherichia coli *(n = 127)**	**31 (24.4%)**	**26 (83.8%)**	**4 (16. 1%)**
Blood (n = 8)	7 (87.5%)	7	-
Urine (n = 91)	9 (9.9%	6	3
W/S (n = 26)	13 (50%)	12	1
*Others (n = 2)	2 (100%)	1	-

***Klebsiella pneumoniae *(n = 91)**	**58 (63.7%)**	**52(89.6%)**	**6 (10.4%)**
Blood (n = 31)	29 (93.5%)	29	-
Urine (n = 32)	11 (34.4%)	5	6
W/S (n = 26)	18 (66.6%)	18	-
*Others (n = 2)	0 (100%)		

***Acinetobacter spp *(n = 39)**	**7 (17.9%)**	**7 (100%)**	**0 (0%)**
Blood (n = 1)	1(100%)	1	-
Urine (n = 4)	2(50%)	2	-
W/S (n = 24)	4(16.6%)	4	-
*Others (n = 10)	0	2	

***Proteus spp *(n = 31)**	**2 (6.4%)**	**2 (100%)**	**0 (0%)**
Blood (n = 1)	0	0	-
Urine (n = 6)	0	0	-
W/S (n = 17)	1(68.6%)	1	-
*Others (n = 7)	1(14.3%)	1	

***Other enterobacteria *(n = 43)**	**12 (27.9%)**	**12 (100%)**	**0 (0%)**
Blood (n = 1)	-	-	-
Urine (n = 17)	3(17.6%)	3	-
W/S (n = 22)	9(40.9%)	9	-
*Others (n = 3)			

***Pseudomonas spp *(n = 46)**	**NA**	**NA**	**NA**
Blood (n = 1)			
Urine ( = 4)			
W/S (n = 30)			
* Others (n = 11)	**NA**	**NA**	**NA**

*Others = Sputum, aspirates and bones chips, n = 377 (inpatients 283, out patients 94), NA = not applicable.

About 95.5% of all GNB were resistant to ampicillin. The rate of resistances to gentamicin, tetracycline, SXT and ciprofloxacin was significantly higher in ESBL-producing organisms than in non-ESBL producers (P = 0.00001; table 2). About 95% of ESBL isolates were found to be resistant to cefepime. A total of 32 (78%) of isolates from ICU (NICU, AICU) produced ESBL and of these, 27(84%) were *Klebsiella pneumoniae*.

**Table 2:** Rate of resistances to SXT, TE, CIP and G among ESBLs and Non ESBL

**Isolates**	**SXT (% R)**	**TE (% R)**	**CIP (% R)**	**G (% R)**
*Acinetobacter spp *(n = 39)	87.2	84.6	43.6	76.9
Non ESBL (n = 32)	65.6	62.5	25	53
ESBL (n = 7)	100	100	43	100

*Escherichia coli *(n = 127)	65.4	59.1	33.1	29.1
Non ESBL (n = 96)	58.3	48.9	23.9	8.3
ESBL (n = 31)	87.1	93.5	61.3	93.5

*Klebsiella pneumoniae *(n = 91)	48.3	74.7	45	68.1
Non ESBL (33)	51.5	48.5	6.1	12.1
ESBL (58)	72.4	89.6	20.7	100

*Proteus spp *(n = 31)	58.4	-	0	16.3
Non ESBL (29)	55.1	-	0	10.3
ESBL (2)	100.0	-	0	100.0

*Other enterobacteria *(n = 43)	72	44.2	21	34.8
Non ESBL (n = 31)	61.3	38.7	12.9	9.6
ESBL (n = 12)	100.0	58.3	41.6	100.0

*Pseudomonas spp (n = 46)*	97.8	93.5	19.6	34.8

SXT = Sulphamethaxazole/trimethoprim, TE = tetracycline, CIP = ciprofloxacin, and G = Gentamicin

## Discussion

There is presently little information on antibiotic susceptibility patterns for Gram negative bacteria in Tanzania, especially in Mwanza. This is the first report on the resistance pattern of gram negatives bacteria in this hospital. Within this institution, treatment is generally given on empirical basis, often not guided by culture results. A total of 377 Gram negative bacteria were recovered from clinical samples, majority of these were from urine and wound swabs. This is due to the large number of these specimens compared to other types of samples collected for microbiological culture. In the present study *Escherichia coli *was the most frequently isolated bacterium and was probably associated with the high proportion of urine specimens examined [16]. *Acinetobacter spp *was commonly recovered from infected wounds and more than 75% were resistant to third generation cephalosporins; with one isolate being resistant to all antibiotics tested. Typical ESBLs production was observed in 17.9% among *Acinetobacter spp*. In other studies ESBL production in *Acinetobacter spp *has been found to range from 20% in India to 54.6 per cent in Korea [17]. Epidemics of *Acinetobacter spp *resistant to cephalosporin and carbepenems have been reported in many surgical centers [18].

In this study most of GNB were resistant to ampicillin with 92.7% of *Escherichia coli *being resistant to this drug. This reflects data previously reported from a study done in Muhimbili whereby more than 80% of *Escherichia coli *were resistant to ampicillin [9]; therefore, the use of this drug is questionable in suspected GNB infection in our setting. In the present study about 43.5% of isolates were resistant to third generation cephalosporins. This prevalence is high and could possibly be a consequence of inappropriate use of these antibiotics at this tertiary hospital without a guide of culture results. A heavy use of antibiotics has been reported to be a risk factor for acquisition of ESBL producing organisms [19]. Several studies have demonstrated the relationship between third generation cephalosporins use and acquisition of ESBL-producing organisms [19-21]. Among all GNB 29.8% of them were found to produce ESBL, an observation which is concordant with findings from studies in South Africa and Asia [3].

Varieties of Gram negatives enteric bacteria were found to produce ESBLs in our hospital. ESBL production among *Escherichia coli *was 24.4%; this is similar to what was observed at Muhimbili National Hospital Tanzania by Bloomberg et al [9]. There was significantly higher isolation of ESBL among *Klebsiella pneumoniae *(63.7%) than among other enteric Gram negative bacteria p = 0.00001. A similar finding has been previously described in a study done elsewhere [5,8,20]. *Klebsiella pneumoniae *was the commonest isolate from ICU, Neonatal ICU and premature unit and significant amount of them were ESBL producers. This finding agrees with those described in more than 75% of previous studies, where the majority of *Klebsiella pneumoniae *isolates were found to produce ESBL [19-21].

The predilection for ESBL production by *Klebsiella pneumoniae *has never been clearly explained. Almost all non-ESBL producing *Klebsiella pneumoniae *isolates have chromosomally mediated SHV-1 β-lactamase [22]. This could also explain why 100% of our *Klebsiella pneumoniae *and *Klebsiella oxytoca *were resistant to ampicillin. In this study other enteric bacteria like *Proteus spp*, *Morganella morganii, Serratia spp*, *Citrobacter spp *and *Salmonella spp *were found to produce ESBL at lower rates, similar to results obtained in other studies else where [1-3].

The prevalence of ESBL is high and poses threat in the treatment of serious infection due to these isolates. Most of ESBL-producing isolates in the present study were significantly resistant to gentamicin, SXT, tetracycline and ciprofloxacin p = 0.00001. Under such circumstances, the only treatment choices are the carbepenems which are expensive and often not available in most centers in our country. Many ESBL genes are on large plasmids, which carry multiple resistance genes [16]; this can explain why most of our ESBL producing organisms were significantly resistant to multiple antibiotics. All the ESBL isolates from this study were sensitive to meropenem and imipenem. Carbepenems have been considered as drugs of choice to ESBL isolates; different studies and clinical trials support the use of these drugs [23,24].

In this study it was found that most of the isolates recovered from blood culture were producing ESBL and this was associated with an increased mortality of those patients. This was also demonstrated in the study from 39 patients at Muhimbili National Hospital, Tanzania [9]. Most of blood stream infections in the present study were from NICU, AICU and Premature unit; these units accounted for 42% of all ESBL isolates in this study. Intensive care units seem to be the epicenter of ESBL-production in the hospital. A similar finding has been demonstrated previously in which more than 40% of hospital's ESBL-producing organisms were recovered from intensive care units [25]. The spread of ESBL in these units can be due to the clonal spread of a single strain or spread of mobile genetic elements among enterobacteriaceae [1]. In our study the majority of patients with ESBLs blood stream infections were successfully treated with meropenem; early detection and early initiation of the appropriate drug was associated with increased survival. Other investigators have also reported similar trend [26,27].

Approximately three quarters of the isolates were from inpatients and of these 35.3% were found to produce ESBL. This was significantly higher than in outpatients p = 0.00001. Some other studies have demonstrated a statistically significant increase in antibiotic resistance in those organisms isolated after 72 hours of admission [27,28]. This suggests that nosocomial acquired organisms are more likely to become ESBL and this is likely to result in treatment failure with empirical use of cephalosporins.

## Conclusion

ESBL isolates are prevalent in our setting and they are multiply resistant to gentamicin, ciprofloxacin, tetracycline and sulphamethaxazole/trimethoprim. Routine detection of ESBL isolates and proper control measures are recommended so that appropriate management can be instituted.

## Competing interests

The authors declare that they have no competing interests.

## Authors' contributions

SEM was principal investigator, participated in design and execution of the work including specimens' collection, microbiological procedures and analysis, interpretation of data and manuscript draft, was the main responsible author. ER and MM participated in planning, microbiological investigation and data analysis. TC and EFL participated in planning and critically revising the manuscript. All authors read and approved the final manuscript.
